# Supplementary material for: Species-Specific Sensitivity and Levels of Beta-D-Glucan for the Diagnosis of Candidemia—A Systematic Review and Meta-Analysis
Source: J Fungi (Basel). 2025 Feb 15;11(2):149. doi: 10.3390/jof11020149 (PMC11856011; doi:10.3390/jof11020149)

Supplementary material for:

Title: Species-Specific Sensitivity and Levels of Beta-D-Glucan for the Diagnosis of Candidemia—A Systematic Review and Meta-Analysis

Nadir Ullah [1], Marco Muccio [1], Laura Magnasco [2], Chiara Sepulcri [1], Daniele Roberto Giacobbe [1,2], Antonio Vena [1,2], Matteo Bassetti [1,2] and Malgorzata Mikulska [1,2*]

Figure S1– Number of Journal articles in the three databases for the selected keywords for the defined time range (2010-2023)

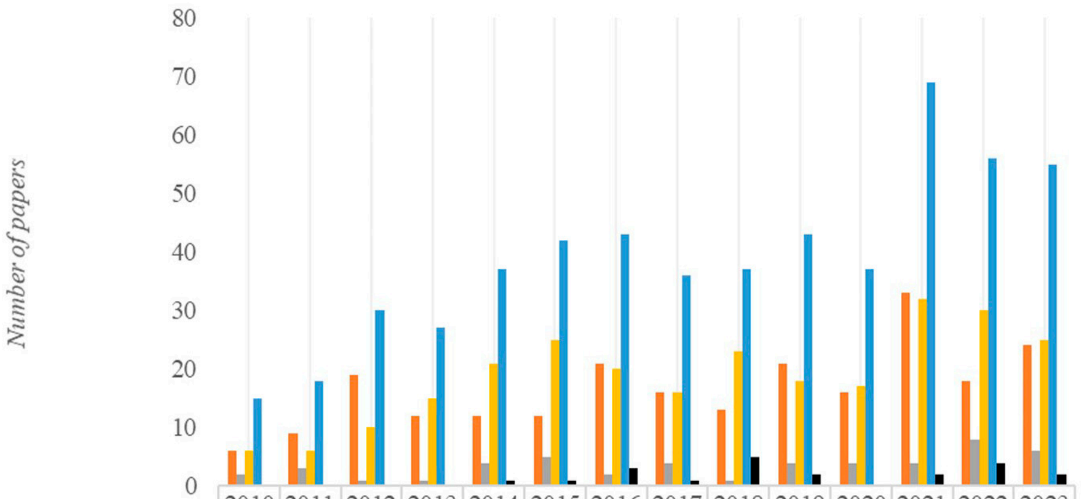

	2010	2011	2012	2013	2014	2015	2016	2017	2018	2019	2020	2021	2022	2023
Science Direct	6	9	19	12	12	12	21	16	13	21	16	33	18	24
Pubmed	2	3	1	1	4	5	2	4	1	4	4	4	8	6
Scopus	6	6	10	15	21	25	20	16	23	18	17	32	30	25
Overall papers	15	18	30	27	37	42	43	36	37	43	37	69	56	55
Included articles	0	0	0	0	1	1	3	1	5	2	0	2	4	2

Table S1– Underlying conditions and clinical characteristics of the patients included in 21 identified studies.

Study ID	Underlying conditions/ clinical characteristics
Träger et al., 2023 [32]	Hemato-oncological= 45.8%, abdominal= 16%, cardiological= 11.4%, pulmonary= 5.4%, immunological= 3.8%, neurological= 4.6%, and other various underlying conditions= 13%.
Lee et al., 2023 [27]	Cardiovascular disease= 43%, HM= 44.1%, solid cancer= 31.2%, diabetes mellitus= 29%, HSCT= 10.8%, CLD= 17.2%, SOT= 17.2%, and CKD= 9.7%.
Mikulska et al., 2022 [25]	Critically ill (ICU)= 100%.
Forster et al., 2022 [36]	Critically ill patients (ICU)= 46.3%, non-ICU= 42.6% and other not-specified.
Corcione et al., 2022, Italy [41]	Diabetes= 26.9%, SOC= 19.2%, cirrhosis= 19.2%, dementia= 15.4%, moderate to severe CKD= 15.4%, CDI= 19.2%, immunosuppressive therapy= 11.5%, atrial fibrillation= 7.7%, hypertension= 51.5%, COPD, 15.4%, CHF= 11.6%, CAD= 7.7%, and cerebral accident= 11.5%.
Kazancioglu et al., 2022 [28]	Diabetes mellitus= 40%, hypertension=66.7%, immunosuppression/malignancy= 20%, CRF= 6.7%, CAD= 46.7%, COPD= 20%, cerebrovascular disease= 26.7%, and surgery= 26.7%.
Esteves et al., 2021 [37]	Cardiovascular disease= 30.9%, diabetes= 29.6%, ARF= 19.7%, CRF= 29.6%, solid tumor= 18.3%, HM= 12.7%, SOT= 18.3%, and HCT= 4.2%.
Farooqi et al., 2021 [26]	Not provided the underlying condition specifically.
Chibabhai et al., 2019 [31]	Oncology (Hematological and solid organ) and Critically ill.
Dichtl et al., 2019 [33]	Critically ill (ICU)= 55%, transplant (SOT and HSCT)= 36%, and other not specified.
Friedrich et al., 2018 [34]	Abdominal surgery= 24.2%, miscellaneous= 21.7%, solid tumor= 20.8%, HM/HSCT= 10%, major surgery= 6.7%, gastrointestinal disease= 5.8%, liver cirrhosis= 5.8%, and Intravenous drug abuse= 5%.
Guitard et al., 2018 [42]	AML= 31.8%, T cell lymphoma= 4.5%, RAEB= 9%, myeloma= 13.6%, multiple myeloma= 4.5%, plasmablastic lymphoma= 4.5%, Hodgkin lymphoma= 9% and myelofibrosis= 4.5%.
Murri et al., 2018 [35]	Diabetes= 6.1%, SOT= 1%, SOC= 30%, dialysis= 1% cirrhosis= 7.1%, CRF=8.1%, obesity= 2%, and septic shock= 9.1%.
Munoz et al., 2018 [40]	Malignancy= 46.6%, diabetes= 43.3%, cardiovascular= 36.6%, gastrointestinal disease= 30%, CLD= 16.6%, and CRD= 20%.

McKeating et al., 2018 [47]	The patients were admitted to different wards, such as ICU, oncology, and surgical ward. The details were not provided.
Son et al., 2017, South Korea [43]	Diabetes= 7%, solid tumor= 67%, rheumatologic disease= 7% and liver cirrhosis= 20%.
Angebault et al., 2016 [38]	Hematology= 58.3%, Critically ill (ICU)= 31.7%, and SOT= 9.7%.
Mikulska et al., 2016 [45]	Chronic renal failure=36.4%, Diabetes mellitus= 30.8, HM= 26.1%, Solid tumor= 45.8%, HSCT= 10.2%.
Leon et al., 2016 [44]	Solid tumor= 36.4%, diabetes mellitus= 36.4%, CRF= 18.2%, and heart failure= 18.2%.
Martínez et al., 2015 [39]	Gastrointestinal disease= 58.1%, solid tumor= 15.6%, liver disease= 19.4%, genito-urinary disease= 22.6%, cardiovascular disease= 19.4%, renal insufficiency= 19.4%, diabetes= 19.4%, hematological disease= 6.5%, lung disease= 9.7%.
Masahiro et al., 2014 [46]	Diabetes mellitus= 33%, Chronic renal failure= 31%, Liver cirrhosis= 3%, HM= 18%, Solid tumors= 36%.

Abbreviations: ARF: acute renal failure; CKD: chronic kidney disease; CDI: Clostridium Difficile infection; COPD: chronic obstructive pulmonary disease; CAD: coronary artery disease; CLD: chronic liver disease; CRF: chronic renal failure; CHF: chronic heart failure; HM: hematologic malignancy; HCT: hematopoietic cell transplantation; HSCT: hematopoietic stem cell transplantation; ICU: intensive care unit; non-ICU: non-intensive care unit; RAEB: refractory anemia with excess blasts; SOT: solid organ transplant; SOC: solid organ cancer.

Table S2–Details of the studies that reported patients admitted to various clinical settings.

Study ID	ICU	Non-ICU	Hematology/ Oncology/ Transplant	Surgery	Medical/ Clinical	General patients Ward or not specified	Brief Description
Lee eta al., 2023 [27]	42	Non	None	5	44	2	Of the total included patients, 42 were admitted to different ICUs, 44 were admitted to the medical ward, and the remaining were admitted to surgery and emergency. Sensitivity of BDG according to different wards/ underlying conditions<br>ICU= 38.09%<br>Medical ward= 45.4%<br>Surgical ward= 40%<br>Emergency= 0%
Träger et al., 2023 [32]	57	None	None	None	None	56	The study compared BDG levels before and at the time of BC sampling.<br>Sensitivity before BC= 62.4%<br>At the time of BC= 78.6%
Forster et al., 2022 [36]	38	35	Non	Non	None	9	Patients were admitted to ICU and non-ICU wards. For the nine patients, the ward of admission was not specified. Sensitivity of BDG according to different wards/underlying conditions<br>ICU= 60.52%<br>non-ICU= 54.28%
Farooqi et al., 2021 [26]	NS	NS	NS	NS	NS	192	The study did not specifically report the ward of admission but reported that 62 days was the stay for patients admitted to ICU, and median BDG levels were 142.15 pg/ml for ICU patients.
Esteves et al., 2021 [37]	30	None	None	11	18	12	Sensitivity of BDG according to different wards/ underlying conditions<br>ICU= 63.3%, (19/30)<br>Surgical ward= 81.1% (9/11)<br>Clinical ward= 61.1% (11/18)<br>For 12 patients ward of admission was not specified, and BDG sensitivity was also not provided. The study also

							reported nine hematological malignancy (HM) patients, seven BDG-positive, 13 solid organ transplant patients (SOT), 10 BDG-positive, and three hematopoietic cell transplantation (HCT), and all three have positive BDG.
Chibabhai et al., 2019 [31]	137	None	21	17	NA	43	A total of 137 patients were admitted to ICU (73 adult's ICU and 64 NICU).<br>Sensitivity of BDG according to different wards/ underlying conditions<br>Adult ICU= 81%<br>NICU= 92%<br>Oncology= 71%<br>Surgical unit= 94%
Dichtl et al., 2019 [35]	66	None	43	None	None	11	The study reported 66 critically ill patients and 43 transplant patients, and in 11 patients wards of admission were not described.<br>Sensitivity of BDG according to different wards/ underlying conditions<br>ICU= 57%<br>Transplant= 57%
Friedrich et al., 2018 [34]	60	None	None	None	None	60	The study compares the BDG performance between the Fungitell and Wako glucan test in candidemia patients. A total of 60 patients were admitted to the ICU, and in 60 patients, the ward of admission was not described. Sensitivity was reported for overall candidemia patients, and the study did not break down these statistics specifically for ICU or other patients.
Munoz et al., 2018 [40]	5	None	None	None	None	25	The study reported 30 candidemia and was classified into complicated and uncomplicated candidemia; nine patients had complicated candidemia and 21 non-complicated candidemia. Out of the total, 25/30 wards of admission were not described and the study did not break down BDG sensitivity statistics specifically for ICU patients.
McKeating et al., 2018 [47]	3	None	1	6	None	None	Of a total of six surgical patients, three were admitted to the urology ward, and three were admitted to the hepatobiliary ward. Three patients were admitted to ICU, and one patient was admitted to the oncology ward.

Mikulska et al., 2016 [45]	None	None	None	None	None	107	The patients were admitted to ICU/Surgery/Medical/Hematology. The study did not provide the real number of patients admitted to each ward.
Angebault et al., 2016 [38]	13	None	28	None	None	None	They reported 49 patients and 41 candidemia. 13 patients were admitted to the Pediatric ICU, 4 patients were SOT, and 24 were admitted to the hematology ward.<br>Sensitivity of BDG according to different wards/underlying conditions<br>Hematology= 66.6% (16/24)<br>SOT= 100% (4/4)<br>Pediatric ICU= 46.1% (6/13)
Martínez et al., 2015 [39]	2	None	4	15	10	None	The study reported 31 candidemia patients admitted to different wards, and the study did not break down BDG sensitivity statistics specifically for ICU or other ward patients. They only reported overall sensitivity.
Masahiro et al., 2014 [46]	7	None	None	None	None	None	The study did not specify the ward of admission. Only reported 7 patients were in ICU. Other wards are not mentioned in the paper.

Abbreviations: BDG: (1,3)-ß-D-Glucan; BC: blood culture: HM: hematological malignancy; HCT: hematopoietic cell transplantation ICU: intensive care unit; NICU: neonatal intensive care unit; NS: Not specified; SOT: solid organ transplant.

Table S3–Summary of all 21 included studies.

Characteristics	n	Percentage %
**Study Design**		
Retrospective single/multicenter	11	52
Prospective single/ multicenter	8	38
Retrospective case-control	2	10
**Clinical wards**		
ICU	3	14
Mixed	12	57
Hematology-oncology wards	1	5
Internal medicine	1	5
Non-ICU	1	5
Not specified	3	14
**Age of the patients**		
Adult	17	81
Adult and pediatric/neonatal	4	19
**BDG assay type**		
Fungitell	15	71
Wako	3+1*	14
Goldstream	2	10
Fungus (1-3)-β-D-Glucan Test (GCT-110T)	1	5
**BDG assay cut-off**		
≥80 pg/mL	18	86
≥11 pg/mL	2	10
≥7 pg/mL	1	5
**Number of candidemia patients**		
>200	1	5
>100	7	33
>50	4	19
<50	9	43
***Candida spp.* distributions**		
C. albicans	18	
C. parapsilosis	18	
C. auris	3	

N. glabrata /Candida glabrata)	17	
P. kudriavzevii (Candida krusei)	12	
C. tropicalis	14	
Other *Candida* species	13	
**Reference standard**		
Blood Culture	21	100

*: One study reported both Fungitell and Wako assay, only Fungitell assay was included in the analysis.

Figure S2– Risk of bias in the included studies (RoB; risk of bias: App; applicability concerns).

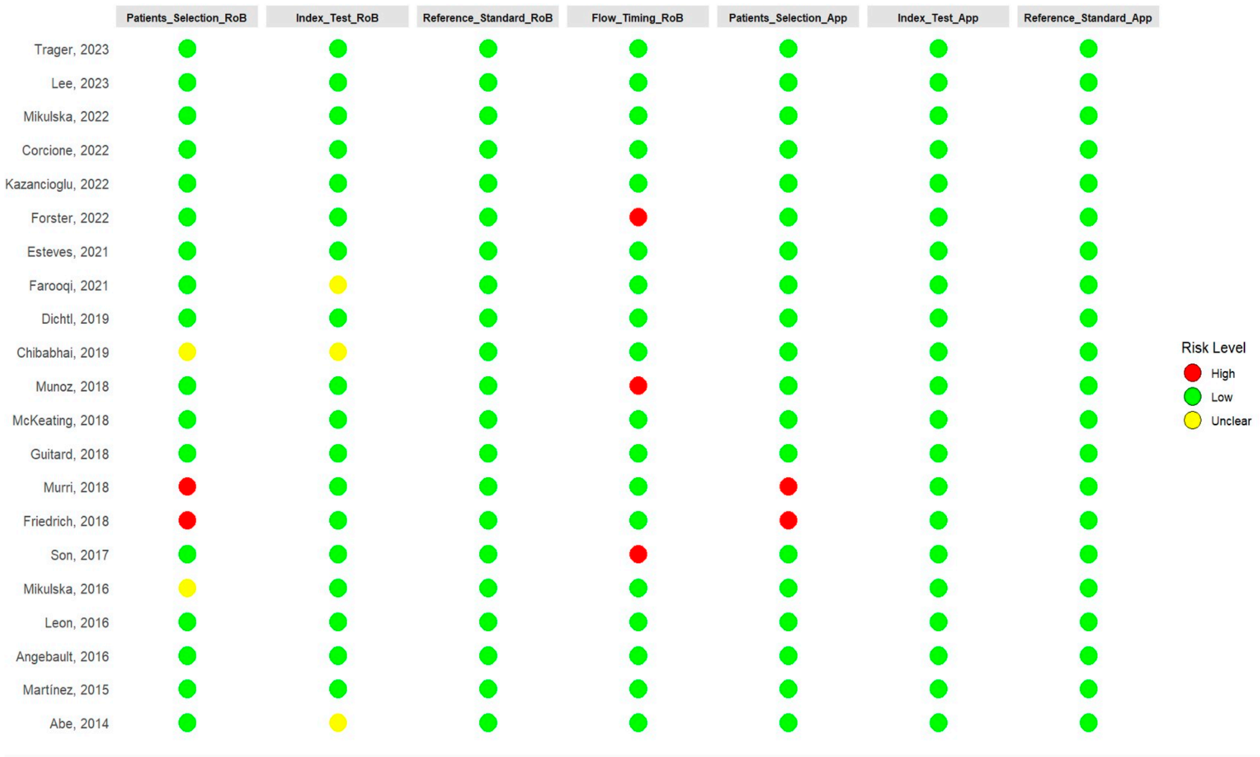

Figure S3–Summary ROC plot of BDG performance analyzed by BDG assay type.

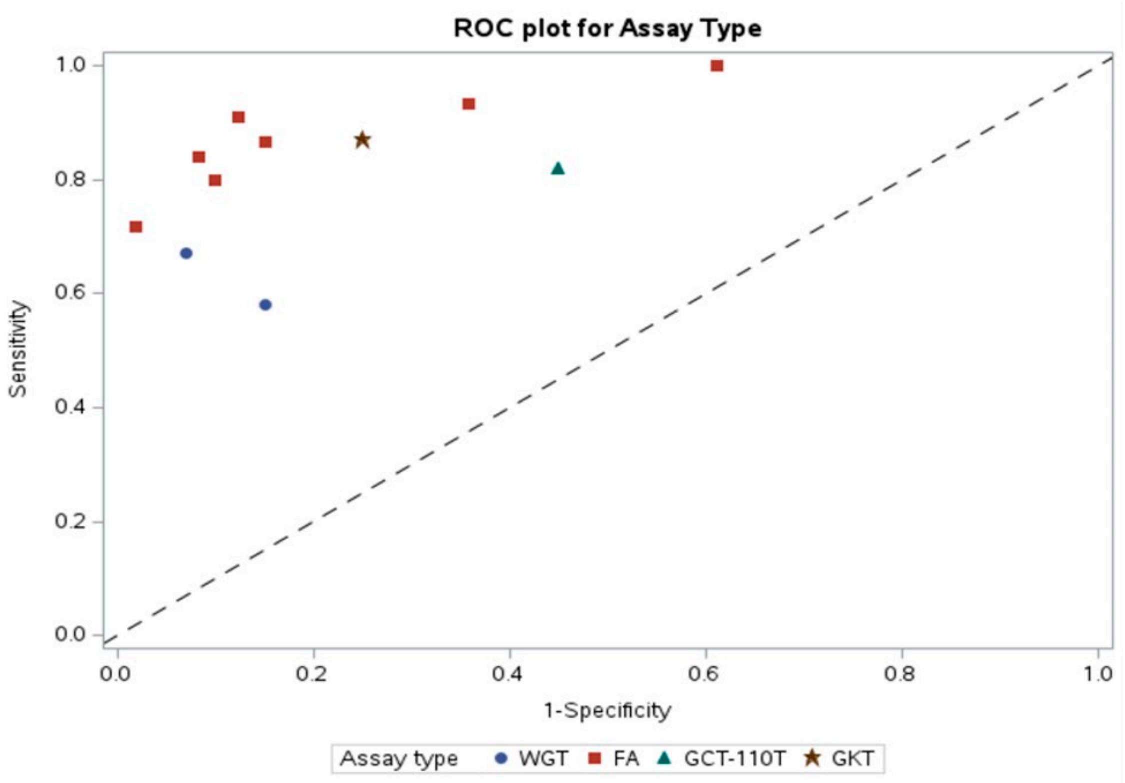

Figure S4–Summary ROC plot of BDG performance analyzed by type of admission ward.

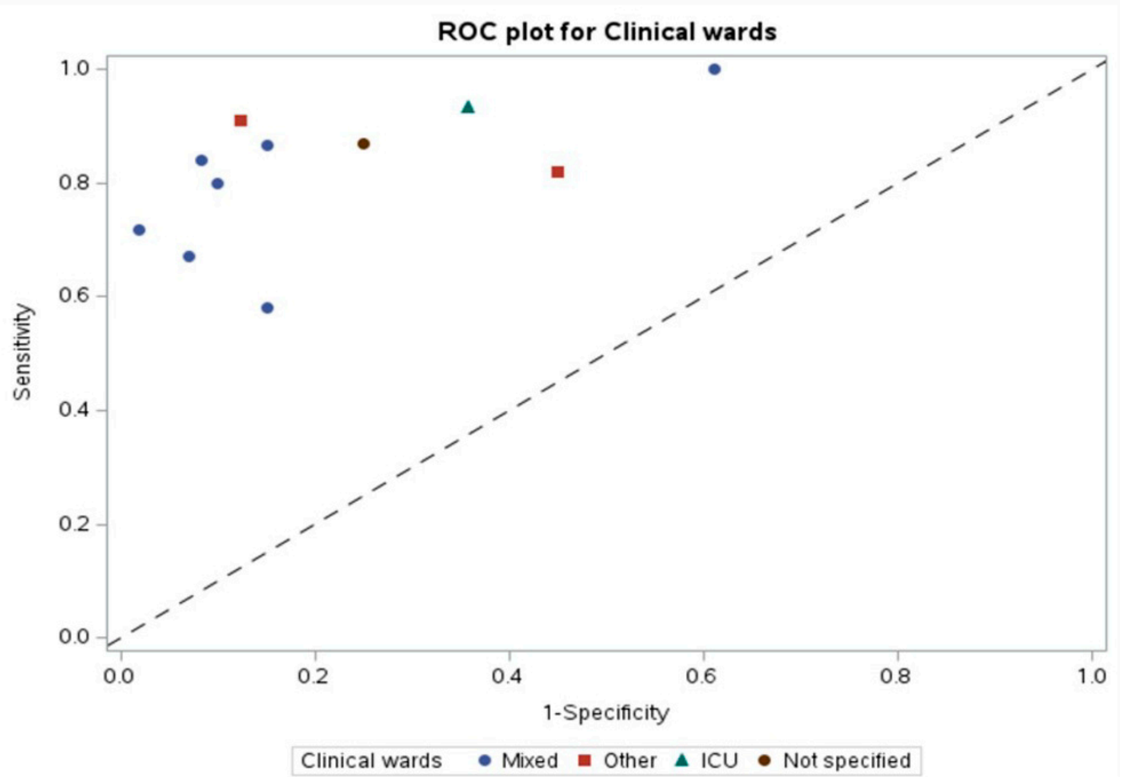

Figure S5– Summary ROC plot of BDG performance analyzed by study design.

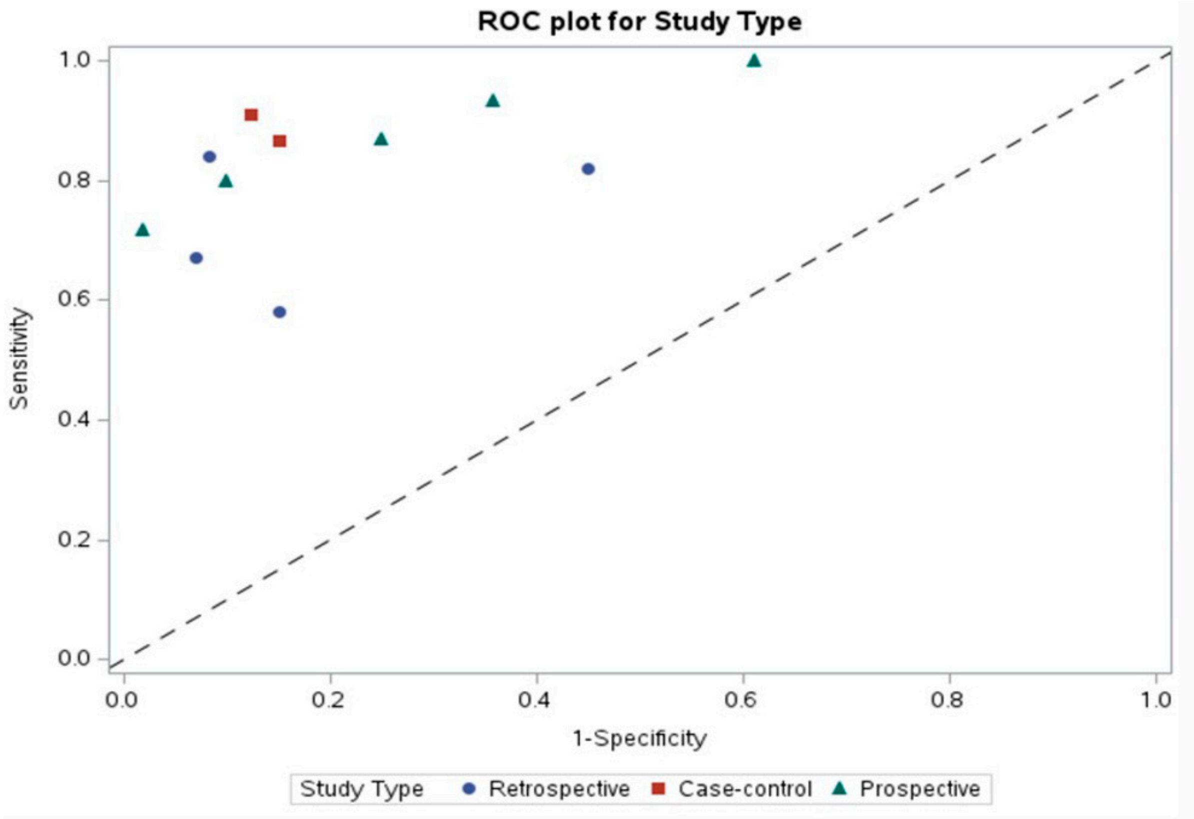

Figure S6–Distributions of *Candida* species reported in 18 included studies (detailed information not available for 3 studies).

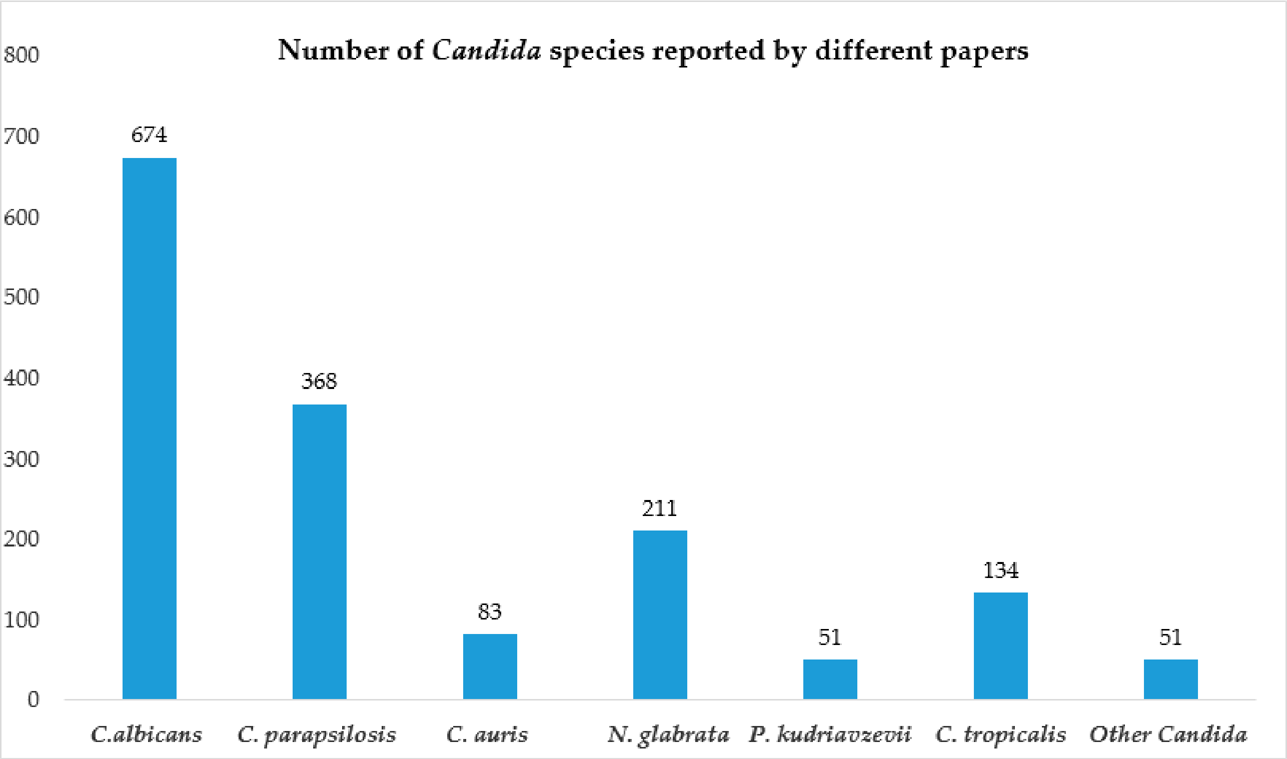

Description of the Figure: In other *Candida* only specified and known species were included in the distribution.

Table S4–Serum species-specific median BDG levels in candidemia patients, with data on BDG assay type, from 09 studies.

Study ID	Species-specific BDG levels pg/mL (in brackets the number of strains of each species)							
	BDG assay type	*C. albicans*	*C. parapsilosis*	*C. auris*	*N. glabrata* (*C. glabrata*)	*P. kudriavzevii* (*C. krusei*)	*C. tropicalis*	Other *Candida* species
Träger et al., 2023 [32]	Fungitell	288 (70)	95 (11)	—	256 (31)	146 (6)	210 (6)	79 (3)
Lee et al., 2023 [27]	Goldstream	328 (28)	232 (7)	—	159 (21)	— (6)	846 (25)	— (6)
Mikulska et al., 2022 [25]	Fungitell	182 (29)	78 (84)	48 (21)	CB (7)	—	CB (4)	CB (1)
Kazancioglu et al., 2022 [28]	Fungitell	461 (5)	165 (5)	—	523 (1)	417 (3)	—	523 (1)
Farooqi et al., 2021 [26]	Fungitell	267 (54)	108 (32)	62 (48)	372 (8)	—	324 (43)	—
Chibabhai et al., 2019 [31]	Fungitell	406 (81)	407 (83)	132 (14)	500 (30)	523 (10)	—	—
Friedrich et al., 2018 [34]	Fungitell	345 (71)	— (10)	—	356 (25)	— (2)	632 (8)	79 (5)*
Friedrich et al., 2018 [34]	Wako	8.4 (71)	— (10)	—	7.5 (25)	— (2)	17.12 (8)	5.9 (5)*
Mikulska et al., 2016 [45]	Fungitell	419 (46)	39 (37)	—	— (10)	— (4)	— (7)	— (3)
Angebault et al., 2016 [38]	Fungitell	392 (16)	109 (4)	—	224 (4)	320 (3)	260 (3)	— (9)

Abbreviations; BDG: (1,3)-ß-D-Glucan; CB: combined BDG levels with other species; —: not reported; * shows BDG levels were available for 5 out of 7.

Table S5–Details of other *Candida* species

Study ID	*Candida* species	Number of *Candida species*	Positive/Negative	Median BDG levels pg/mL
Lee et al., 2023 [27]	*C. lusitaniae* (now *Clavispora lusitaniae*)	4	2/2	—
	C. guilliermondii (now *Meyerozyma guilliermondii*)	2	0/2	—
Träger et al., 2023 [32]	*C. lusitaniae*	3	1/2	79
Mikulska et al., 2022 [25]	*C. lusitaniae*	1	CS	CB
Forster et al., 2022 [36]	*C. lusitaniae*	1	Not available	Not available
	M. guilliermondii	1	Not available	Not available
	C. kefyr (now *Kluyveromyces marxianus)*	1	Not available	Not available
Kazancioglu., et al 2022 [28]	*C. famata* (now *Debaryomyces hansenii*)	1	1/0	523
Farooqi., et al 2021 [26]	Other/not specified	7	6/1	221.73
Esteves., et al 2021 [37]	*M. guilliermondii*	1	Not available	Not available
	C. norvegensis (now *Pichia norvegensis*)	1	Not available	Not available
Dichtl et., 2019 [33]	*M. guilliermondii*	4	2/2	Not available
	Other/not specified	4	3/1	Not available
Friedrich et al., 2018 [34]	*C. lusitaniae*	1	Not available	79
	M. guilliermondii	3	Not available	88
	C. dubliniensis	2	Not available	Not available
	Candida fabianii (now *Cyberlindnera fabianii*)	1	Not available	7
Guitard et al., 2018 [42]	*C. dubliniensis*	1	0/1	Not available
	K. marxianus	4	2/1+B	Not available
	C. lusitaniae	1	B	Not available
Angebault et al., 2016 [38]	*C. lusitaniae*	3	1/2	<80
	M. guilliermondii	4	0/4	<80
	P. norvegensis	2	0/2	<80
	Hyphopichia burtonii	1	0/1	<80
	Kodamaea ohmeri	1	0/1	<80
Mikulska., et al 2016 [45]	*C. lusitaniae*	1	CS	CB
	M. guilliermondii	1	CS	CB
	K. marxianus	1	CS	CB
Masahiro., et al	*C. lusitaniae*	2	1/1	Not available

2014 [46]	*M. guilliermondii*	2	2/0	Not available
	Other/not specified	4	3/1	Not available

Abbreviations: B: Borderline; CS: Combined sensitivity with other *Candida species;* CB: Combined BDG levels with other *Candida* species

Figure S7–Pooled sensitivity of *C. albicans* (A. all assays vs. B. Fungitell only).

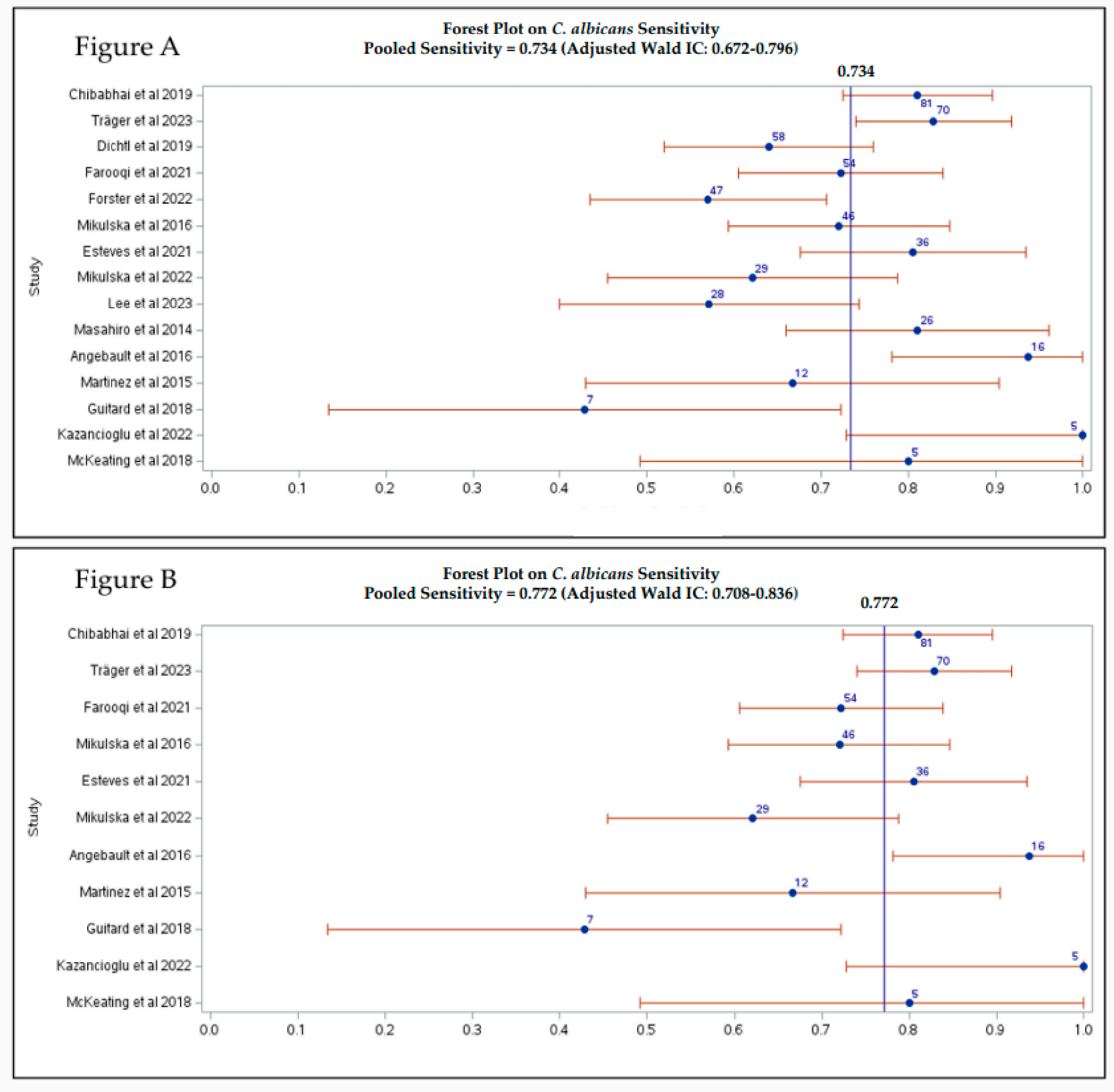

Figure S8–Pooled sensitivity of *C. parapsilosis* (A. all assays vs. B. Fungitell only).

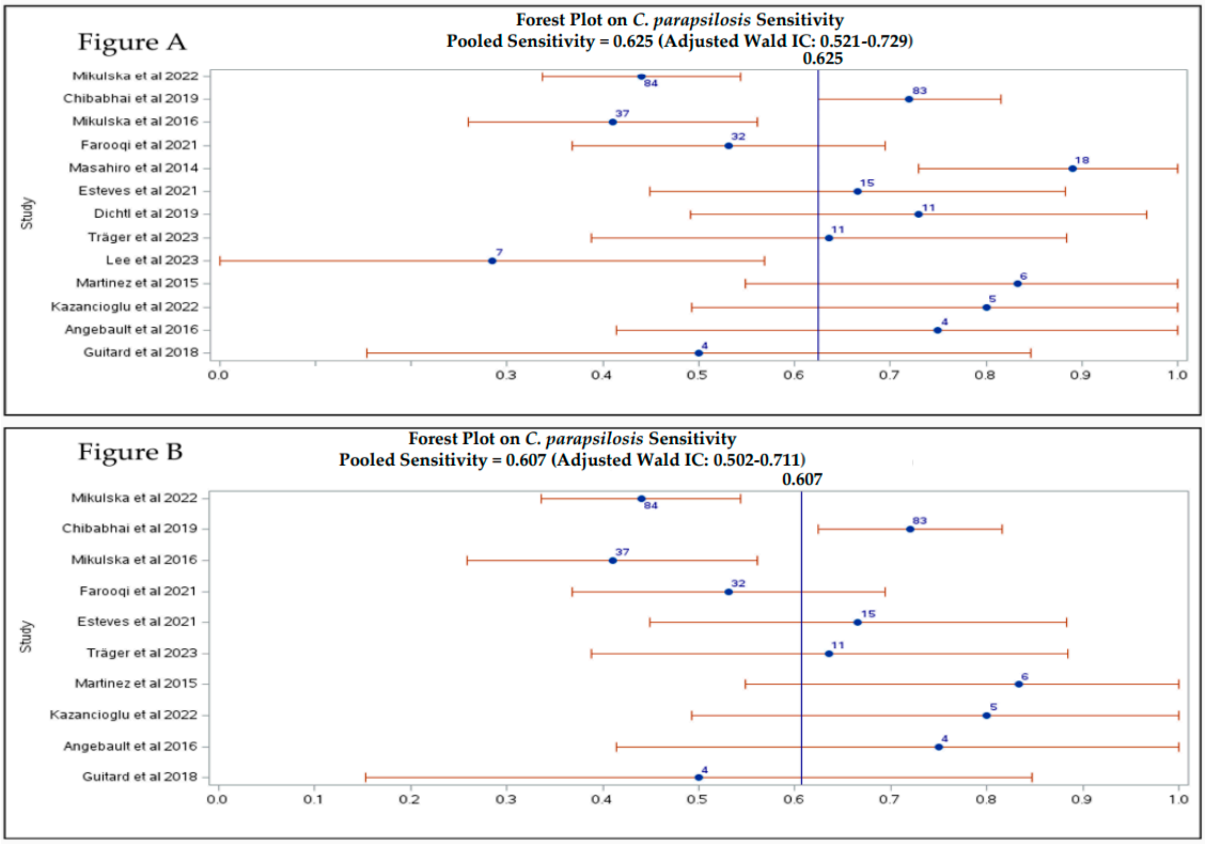

Figure S9–Pooled sensitivity of *C. auris* (Fungitell only).

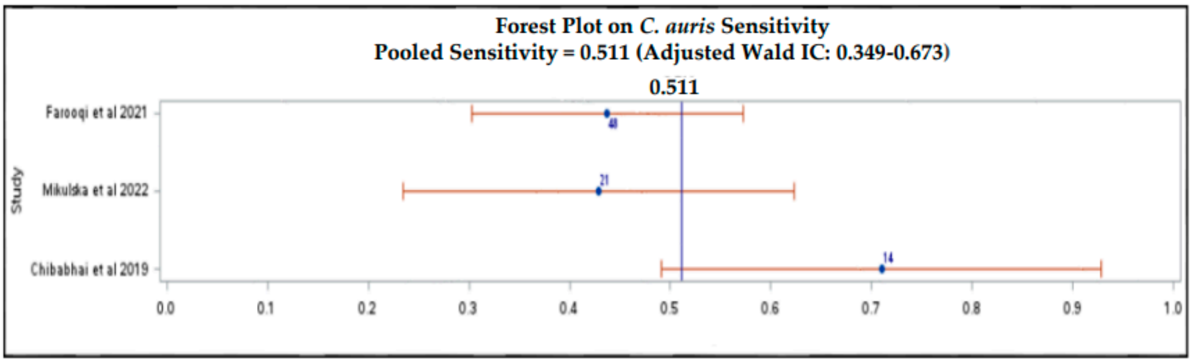

Figure S10–Pooled sensitivity of *N. glabrata (previously C. glabrata)* (A. all assays vs. B. Fungitell only).

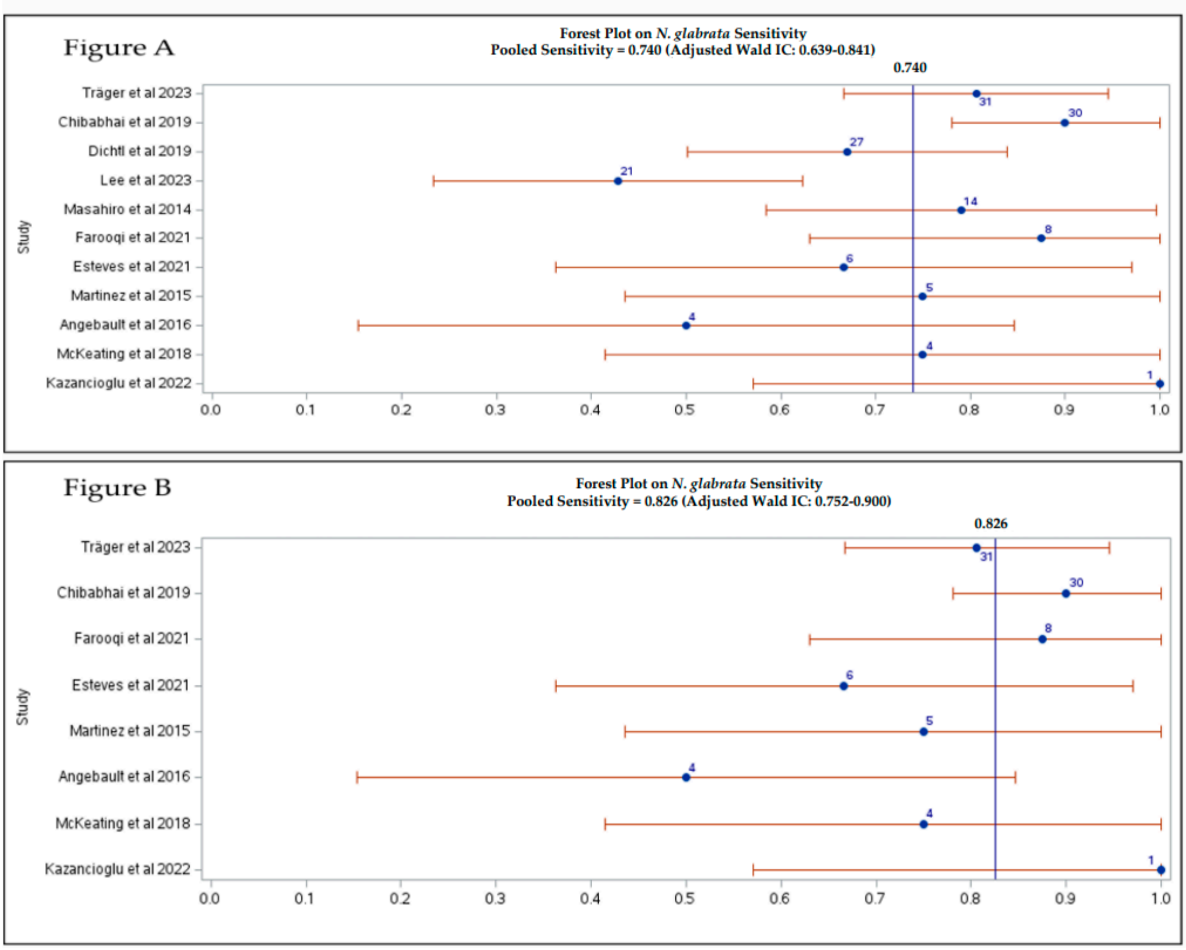

Figure S11–Pooled sensitivity of *P. kudriavzevii* (*previously C. krusei*) (A. all assays vs. B. Fungitell only).

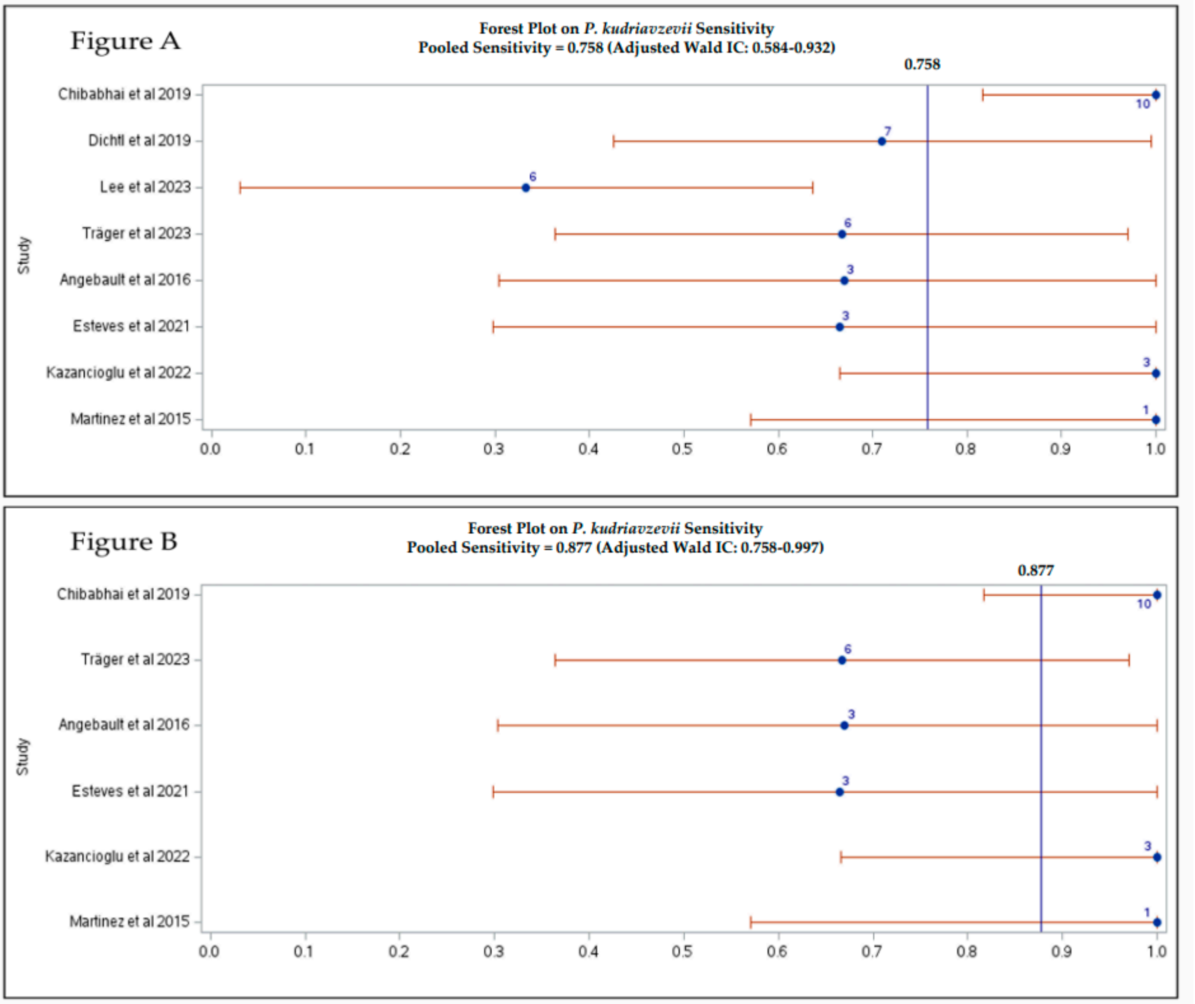

Figure S12–Pooled sensitivity of *C. tropicalis* (A. all assays vs. B. Fungitell only).

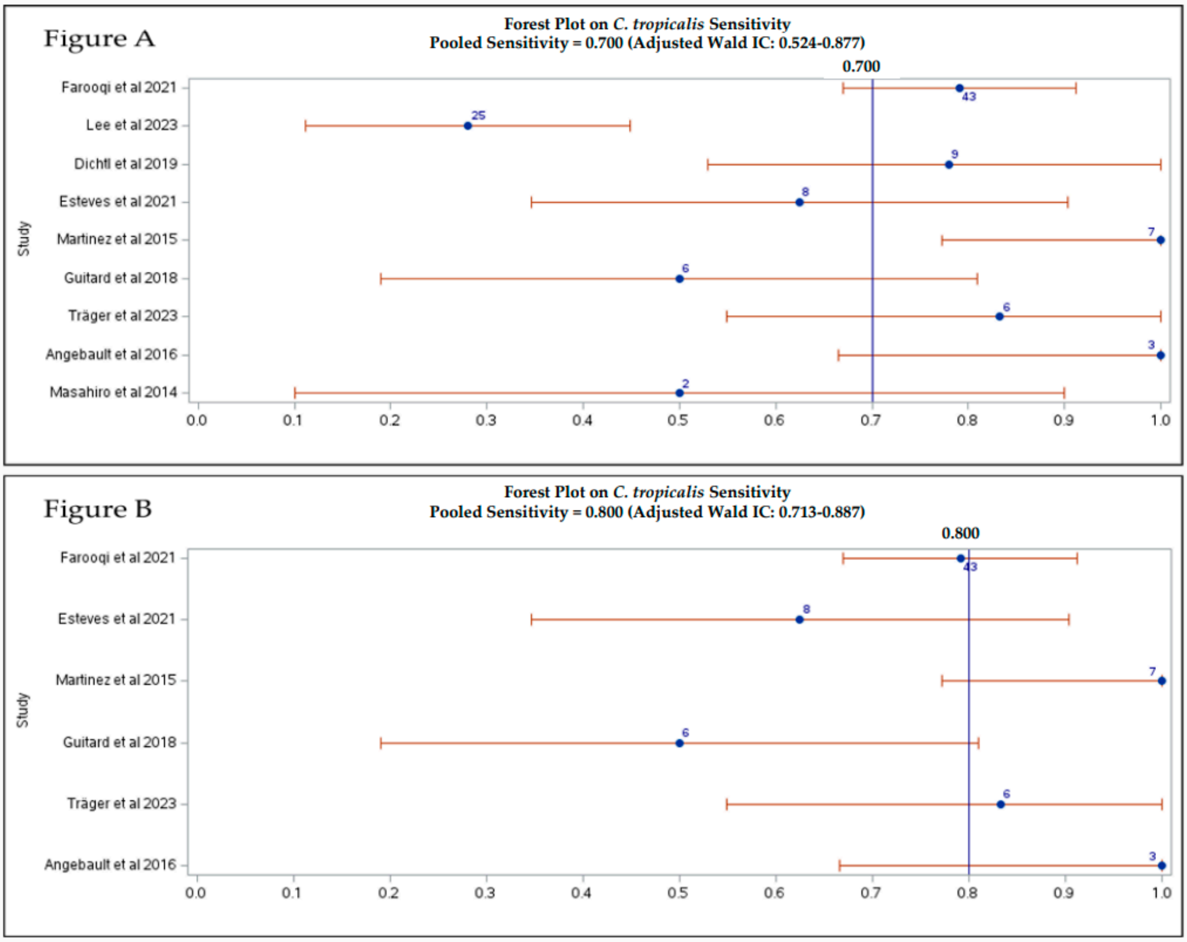

Figure S13– Pooled sensitivity of other *Candida* species (A. all assays vs. B. Fungitell only).

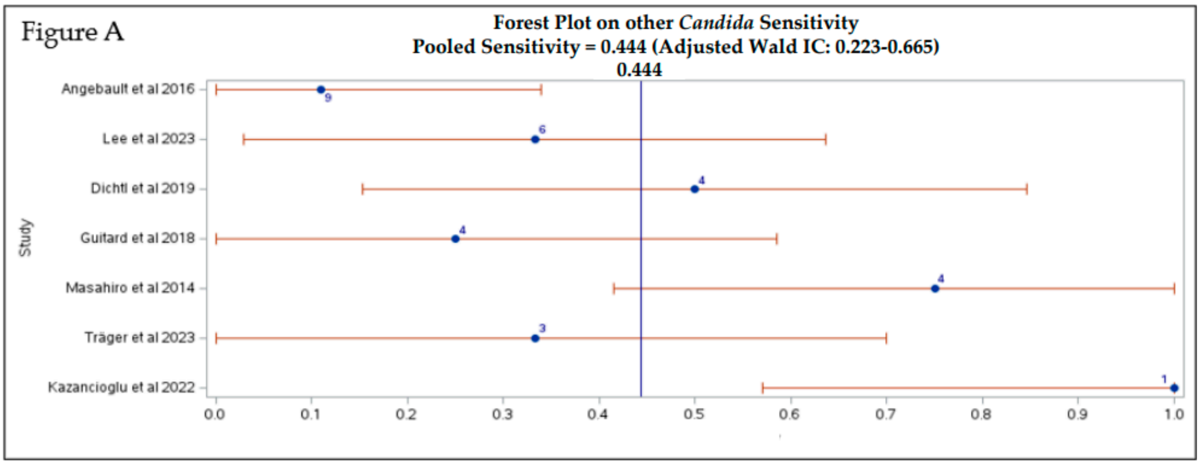

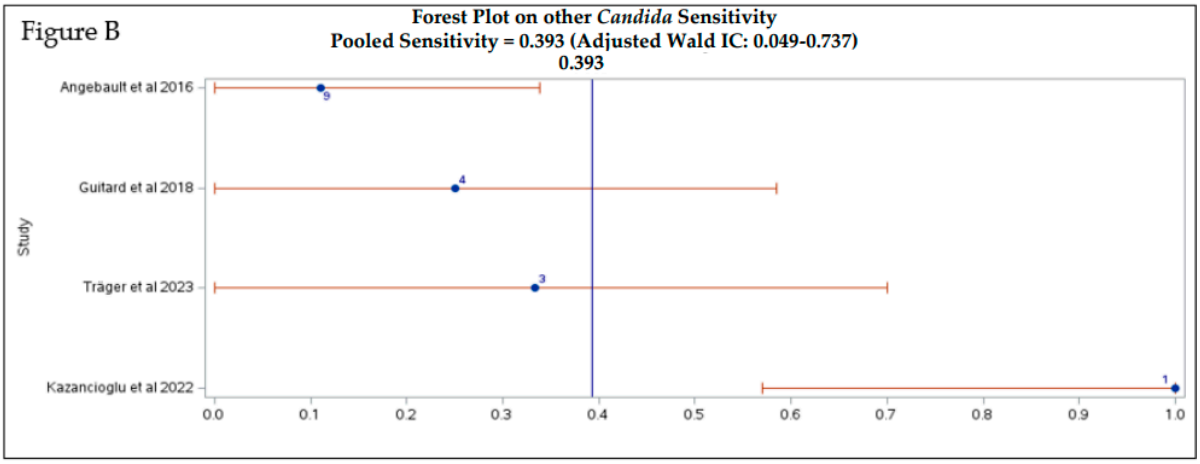

Supplement: Supplementary file 1 [file jof-11-00149-s001.zip › jof-3441545-supplementary.pdf]
